# Splenic switch-off, a potential novel marker of lack of adenosine response: prevalence and measurement reproducibility

**DOI:** 10.1186/1532-429X-17-S1-P122

**Published:** 2015-02-03

**Authors:** Marinos Koulouroudias, Alice Lighton, Filip Zemrak, Charlotte Manisty, James Moon, Ceri Davies, Redha Boubertakh, Mohammed Y Khanji, Ian S Stone, Mark Westwood, Neha Sekhri, Steffen E Petersen

**Affiliations:** Barts and the London School of Medicine and Dentistry, London, UK; The Heart Hospital, London, UK; Centre for Advanced Cardiovascular Imaging, Willam Harvey Research Institute, Queen Mary University of London, London, UK; Barts and The London National Institute for Health Research Biomedical Research Unit, London, UK

## Background

The sensitivity of adenosine stress perfusion CMR scans is reduced by inadequate response to adenosine. This can be due to a variety of environmental and pharmacological factors, including recent caffeine intake. Manisty et al (2014) observed that splenic blood flow is attenuated by adenosine, as the splanchnic circulation contains vasoconstrictor adenosine receptors, and may provide a simple visual marker of adequate pharmacological stress. The aim of this study was to validate measurement of splenic switch-off (SSO) in both a quantitative and qualitative manner and examine its prevalence in an East London tertiary cardiac centre.

## Methods

We examined 503 negative cardiac magnetic resonance (CMR) adenosine perfusion scans for a splenic switch-off (SSO) by visual assessment. All scans were analysed by two independent observers. Disagreements between observers in the qualitative measure were resolved by a third observer. SSO is a graded and transitory response; therefore, we defined the filling of the left ventricle as time zero to ensure synchronous comparison of rest and stress scans. In addition, 252 scans were analysed quantitatively by the two observers using CVI42. The ratio between the brightest mean intensity of a region of interest between stress and rest images, adjusted for baseline intensity, gave a single parameter, the spleen intensity ratio (SIR).

## Results

We were able to assess SSO in 492 out of 503 scans, and 53 (11%) had no SSO. Inter and intra-rater agreement for qualitative measurements was assessed using Cohen's kappa. Inter-rater agreement was excellent with κ=0.81*.* Intra-rater agreement was also good, with κ=0.70. For quantitative measurements, the inter-class correlation coefficient between observers was 0.94, and the Bland-Altman plot shows good agreement across a range of SIRs without systematic bias for a random sample of cases (bias=0.00248, limits of agreement=-0.162 - 0.157).

Using visual assessment as the gold standard, ROC analysis showed that the optimal threshold for SIR as an indicator of SSO was 0.40 (sensitivity = 82.5%, specificity=92.3%, AUC=0.91).

## Conclusions

SSO is a consistently measurable phenomenon and can be assessed visually. Quantitative measurement could be used for resolution of unclear cases and is a reproducible measure. A quantitative measure could be automated and included in future software packages. The prognostic value of SSO as a surrogate of adequate stressing can only be inferred after the acquisition of outcome data.

## Funding

AL recieved a Rod Flower scholarship from Barts and the London School of Medicine and Dentistry.

**Figure 1:**
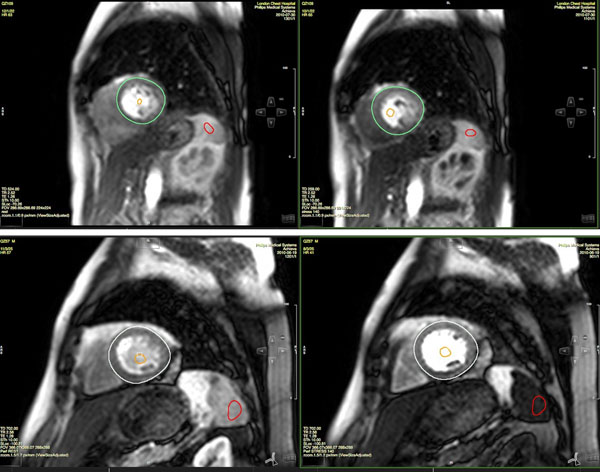
**A and B show rest and stress scans respectively, from a patient with no SSO. C and D show rest and stress scans from a patient with SSO.** A ROI has been selected on the spleen in each case for quantitative analysis.

**Figure 2:**
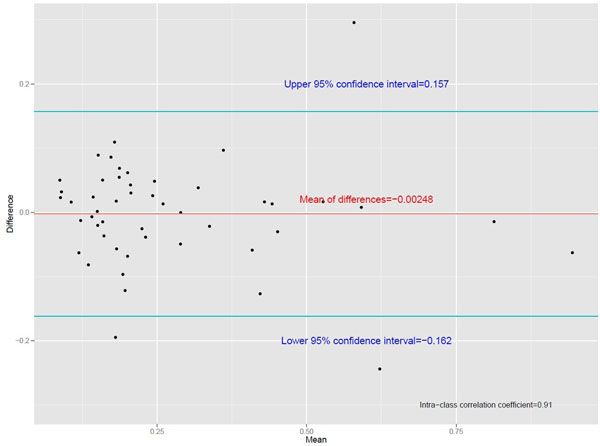
A Bland-Altman plot for a random sample of 50 cases.

